# Vascularized Muscle Flap to Reduce Wound Breakdown During Flexible Electrode-Mediated Functional Electrical Stimulation After Peripheral Nerve Injury

**DOI:** 10.3389/fneur.2020.00644

**Published:** 2020-07-21

**Authors:** Malia McAvoy, Joshua C. Doloff, Omar F. Khan, Joseph Rosen, Robert Langer, Daniel G. Anderson

**Affiliations:** ^1^Harvard-MIT Division of Health Sciences and Technology, Harvard Medical School, Massachusetts Institute of Technology, Boston, MA, United States; ^2^Department of Biomedical Engineering, Translational Tissue Engineering Center, Wilmer Eye Institute, Johns Hopkins University School of Medicine, Baltimore, MD, United States; ^3^Department of Materials Science and Engineering, Institute of NanoBioTechnology, Johns Hopkins University, Baltimore, MD, United States; ^4^Institute of Biomaterials and Biomedical Engineering, University of Toronto, Toronto, ON, Canada; ^5^Department of Immunology, University of Toronto, Toronto, ON, Canada; ^6^Dartmouth-Hitchcock Medical Center, Geisel School of Medicine, Lebanon, NH, United States; ^7^David H. Koch Institute for Integrative Cancer Research, Massachusetts Institute of Technology, Cambridge, MA, United States; ^8^Department of Chemical Engineering, Massachusetts Institute of Technology, Cambridge, MA, United States; ^9^Institute for Medical Engineering and Science, Massachusetts Institute of Technology, Cambridge, MA, United States; ^10^Department of Biomedical and Materials Science Engineering, Translational Tissue Engineering Center, Wilmer Eye Institute and the Institute for NanoBioTechnology, Johns Hopkins University School of Medicine, Baltimore, MD, United States

**Keywords:** peripheral nerve injury, functional electrical stimulation, electrode, wound, vascularized flap

## Abstract

The success of devices delivering functional electrical stimulation (FES) has been hindered by complications related to implants including skin breakdown and subsequent wound dehiscence. Our hypothesis was that a vascularized muscle flap along the dorsal surface of an epimysial electrode would prevent skin breakdown during FES therapy to treat atrophy of the gastrocnemius muscle during peripheral nerve injury. Resection of a tibial nerve segment with subsequent electrode implantation on the dorsal surfaces of the gastrocnemius muscle was performed on ten Lewis rats. In five rats, the biceps femoris (BF) muscle was dissected and placed along the dorsal surface of the electrode (Flap group). The other five animals did not undergo flap placement (No Flap group). All animals were treated with daily FES therapy for 2 weeks and degree of immune response and skin breakdown were evaluated. The postoperative course of one animal in the No Flap group was complicated by complete wound dehiscence requiring euthanasia of the animal on postoperative day 4. The remaining 4 No Flap animals showed evidence of ulceration at the implant by postoperative day 7. The 5 animals in the Flap group did not have ulcerative lesions. Excised tissue at postoperative day 14 examined by histology and *in vivo* Imaging System (IVIS) showed decreased implant-induced inflammation in the Flap group. Expression of specific markers for local foreign body response were also decreased in the Flap group.

## Introduction

During a peripheral nerve injury, the denervated skeletal muscle undergoes an inevitable course of atrophy leading to the muscle being replaced by fibrous connective tissue and fat. Even with optimal medical and surgical management of peripheral nerve injury, muscle function is only partially restored (1). Poor functional recovery is due to the progressive fall in regenerative capacity of neurons with increasing time and distances (2). Sectioned nerves regenerate at a rate of 2-3 mm/day, so large gap peripheral nerve injuries > 5 cm can require approximately over 1 year to heal (3).

Devices delivering functional electrical stimulation (FES) have been developed to prevent muscle atrophy and enhance functional recovery during peripheral nerve injury (4, 5). Electrical stimulation of the proximal nerve stump accelerates nerve outgrowth across the injury site but the distal denervated muscles for extended periods of time become atrophied, diminishing the potential for optimal functional recovery (6–10). Direct electrical stimulation of the denervated muscle may reduce muscle atrophy and increase the number of functional motor units available for recovery (4, 11–15). FES upregulates the production of vascular endothelial growth factor and insulin-like growth factor by mesenchymal stroma cells (16), which are important in myocyte regeneration (17). By increasing myogenic precursor cell proliferation and facilitating fusion with mature myofibers, FES improves the regenerative capacity of skeletal muscle (18, 19).

There are three basic locations where electrodes may be applied for FES: on the surface of the skin (transcutaneous electrodes), embedded within the muscle (intramuscular electrodes) or along the surface of the muscle (epimysial). Transcutaneous electrodes apply the current to a larger region stimulating more muscle groups but also while decreasing current density. Surgical placement of intramuscular electrodes causes damage and trauma to the muscle tissue. Epimysial electrodes may allow for targeted treatment of muscles while minimizing the invasiveness of the electrode itself. Because of the combination of current density and minimized invasiveness, our group has focused on the development of epimysial interventions (5, 20–22).

The reality of implantable epimysial electronics has been hindered by the rigidity of electronic materials (23). Silicon is often used as a substrate for electrodes due to its well-known electrical properties (24). Such hard, electronic materials cause permanent damage of tissue and inflammatory response due to micro motion between the stiff electrode and the underlying muscle. Flexible polymers such as polyimide are being increasingly used as substrates to provide adequate conformability along the muscle surface, establishing a uniform electrode-tissue interface, while tolerating strain to prevent micro motion-related damage (25). However, placement of the electrode directly under the wound may also lead to motion-related damage of the fascial and dermal layers causing ulceration then breakdown of the wound and finally exposure of the implant and infection.

Much work has been dedicated to modifying electrode design to minimize complications although the surgical technique itself has not yet been explored. The development of new surgical techniques may prevent implant-related complications. Vascularized muscle flaps provide a soft-tissue cover and introduce fresh blood supply to the ischemic and contaminated wound aiding in tissue healing and resolution of infection (26). They have been successfully used in a wide range of clinical settings to salvage exposed implants (8, 26, 27). Prophylactic muscle flaps have also been reported in vascular surgery of the femoral vessels in which patients receiving a flap during the initial surgery experienced significantly fewer groin wound complications (28). However, the use of vascularized muscle flaps to prevent skin breakdown in high-risk surgeries involving implantation of epimysial sensors and electrodes, has not been reported. Furthermore, the vascularized muscle flap covering the dorsal surface of the epimysial electrode secures the electrode to the underlying denervated muscle allowing for optimal transfer of electrical current. The rat biceps femoris (BF) muscle has been previously described as a reliable musculocutaneous flap with consistent perforator vasculature (6).

Our hypothesis was that a vascularized muscle flap along the dorsal surface of an epimysial electrode would prevent skin breakdown leading to wound dehiscence during FES therapy to treat atrophy of the gastrocnemius muscle during acute peripheral nerve injury while the nerve is being regenerated. Specifically, we aimed to prevent skin damage due to the presence of an implanted electrode underneath the skin. Because the FES is being delivered to the denervated gastrocnemius muscle in the setting of acute peripheral nerve injury, there is some muscle contraction and thus there is motion of the electrode underneath the skin which may cause further skin ulceration. Our proposed solution is to use the BF muscle as a vascularized flap over the dorsal surface of the electrode. We compared rates of skin ulceration and implant-related tissue among rats with the vascularized BF muscle flap and those without the flap during delivery of FES to the denervated gastrocnemius muscles. These 2 groups were compared according to incidence of wound-related complications, histological and immunofluorescence analysis and *in vivo* quantification of inflammation using fluorescent whole animal imaging.

## Materials and Methods

### Electrode Specifications

The fabrication of this polyimide-based electrode has been published previously in detail (5). Briefly, the electrodes consist of two 5 μm layers of polyimide (PI 2674, HD Microsystems, Parlin, New Jersey), a base layer and a top layer. Between these two layers are a 10 nm Titanium (Ti) adhesion layer and a 500 nm Gold (Au) layer patterned into a design with serpentine wires via standard photolithography techniques. The polyimide layer on the base layer, the side of the electrode interfacing with the underlying denervated gastrocnemius muscle, was etched to expose the gold contact pads which serve to transfer current from the electrode to the muscle. The size of each electrode is a 1 cm by 1 cm square. This electrode design offers low electrode impedance and high charge injection capacity while maintaining flexibility and miniaturized scale.

### Animals

All animal studies were performed at the Massachusetts Institute of Technology's Division of Comparative Medicine. All procedures and studies conducted were approved by the Massachusetts Institute of Technology's Institutional Animal Care and Use Committee (MIT IACUC), in accordance with the guidelines of the Animal Care and Use Review Office (ACURO) of the U.S. Army Medical Research and Development Command (USAMRMC) Office of Research Protections (ORP) (protocol number 0714-080-20) and complied with all applicable local, state and federal regulations. These studies used female Lewis Rats (*n* = 10, age: 10–14 weeks, weight 235–264g, Charles River, Wilmington, MA). The sample size for this pilot study was determined based on prior literature review of similar studies detailing new experimental flap models in rats (29–32) where *n* = 5 was used for each animal group and as part of a discussion with veterinarians at the MIT Division of Comparative Medicine in an effort to minimize the number of areas undergoing this new procedure with unknown morbidity and mortality.

Anesthesia was induced and maintained with inhaled isoflurane (3.0%) in oxygen. Preoperative care included administration of pre-emptive analgesia subcutaneously at the following doses: buprenex (0.03 mg/kg) and meloxicam (1.0 mg/kg). Postoperative care included 24 h checks, buprenex (0.03 mg/kg) administration at 8-h intervals and meloxicam (1.0 mg/kg) administration every 24 h for 72 h. The animals were euthanized on postoperative day 14.

### Surgical Procedure

#### No Flap Procedure

**Figure 1:**
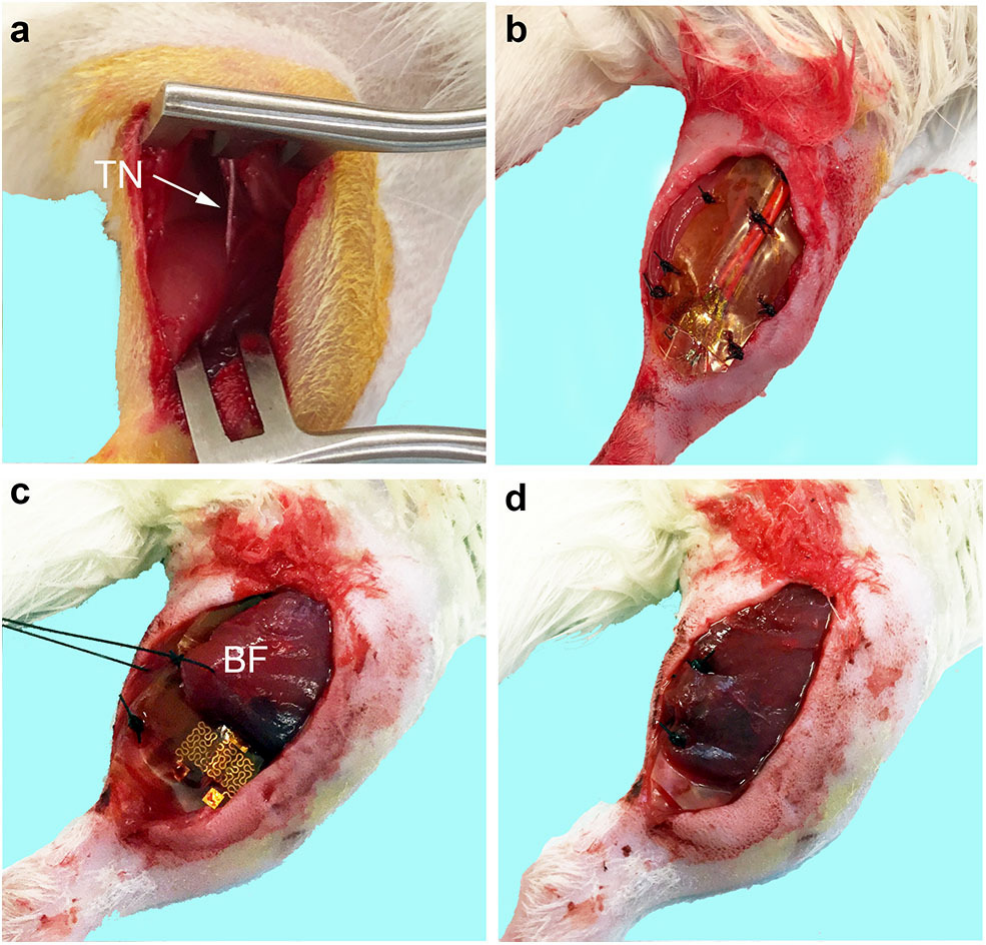
Biceps femoris muscle flap procedure for epimysial electrode implant. **(a)** The tibial nerve (TN) was identified and resected distally and proximally to remove a nerve segment of >1.5 cm in length. **(b)** Flexible electrode implanted onto the dorsal surface of the gastrocnemius muscle without the flap. **(c)** Biceps femoris (BF) muscle was bluntly dissected and used as a vascularized flap over the dorsal surface of the electrode. **(d)** Flap is fixed over the electrode with interrupted 4-0 Nylon and the wound is closed.

Generation of the critical peripheral nerve injury model and implantation of the flexible electrode along the dorsal surface of the gastrocnemius muscle has been described and implemented previously (5). A 2 cm skin incision was made using a scalpel along the dorsal surface of the right hindlimb parallel with the tibia. The BF muscle was bluntly dissected to reveal the sciatic nerve within the posterior fossa. The tibial nerve was identified as the largest and most central branch and the nerve was resected distally (Figure 1a). The tibial nerve was resected to remove a nerve segment no less than 1.5 cm in length. The polyimide-based electrode was sutured to the dorsal surface of the gastrocnemius muscle (Figure 1b) and the wires were tunneled subcutaneously to a 0.5 cm incision made on the scalp. The distal ends of the wires were secured to the scalp using 2 interrupted sutures securing the scalp tightly around the wire and wound clips.

#### Flap Procedure

The same critical peripheral nerve injury and electrode implantation procedure was performed as with the No Flap method. Then the BF muscle was bluntly dissected from its medial and posterior border without injuring the terminal perforating branches of the femoral artery or the motor branches to the BF muscle arising from the gluteal nerve. The BF muscle was sharply dissected along the lateral border and rotated to cover the electrode. The vascular pedicle to the BF muscle was carefully preserved. The distal end of the BF muscle flap was secured to the gastrocnemius muscle using 4-0 Nylon sutures (Figures 1c,d). The skin layer was closed all using interrupted 4-0 Nylon.

### Functional Electrical Stimulation

All animals (*n* = 10) with electrode implants were stimulated 5 times per week beginning on postoperative day 2 and ending on postoperative day 13 for 45 minute sessions per day using 400 ms of 100 Hz frequency, 200 μs per phase biphasic pulses followed by 6 seconds of rest. Stimulus amplitudes were adjusted to maintain a strong contraction evident visually and/or by palpation between 2 and 5 mA as described previously (4, 5, 33). The rats were anesthetized using 3% isoflurane in oxygen and maintained at the same rate. Visual or palpable contractions were evident throughout the entire duration of therapy.

### Postoperative Course and Gross Observation

**Figure 2:**
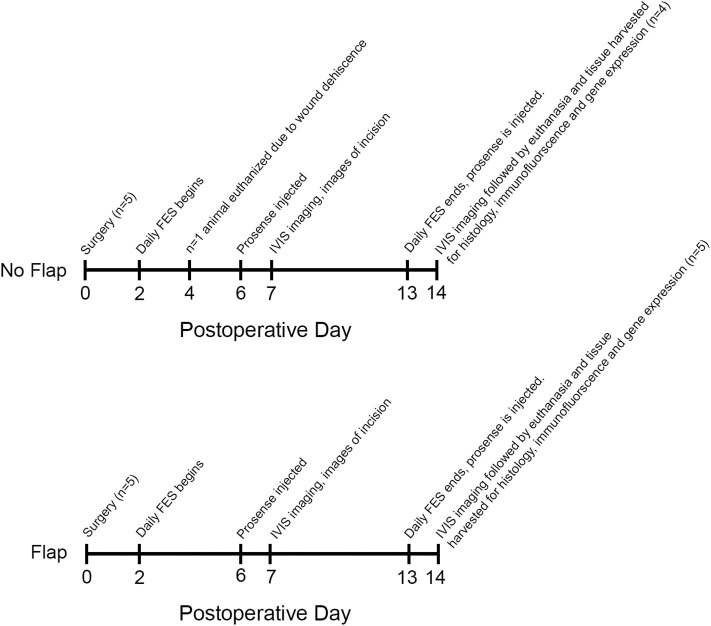
Timeline from surgery until tissue harvesting on postoperative day 14. One animal in the No Flap group was euthanized on postoperative day 4 due to wound dehiscence. On postoperative day 7, IVIS imaging was performed. On postoperative day 14, IVIS imaging was repeated and all animals were euthanized. Tissue surrounding the electrode including gastrocnemius muscle, biceps femoris flap (if present) and overlying skin was harvested for histology, immunofluorescence and gene expression.

A timeline of events from surgery (postoperative day 0) until euthanasia (postoperative day 14) is provided in Figure 2. Daily weights, photographs and visual inspections were performed on each rat. Signs of skin ulceration or wound dehiscence were recorded.

### *In vivo* Imaging System (IVIS)

Fluorescent whole animal imaging after absorption of ProSense 750 FAST (NEV11171, PerkinElmer, Waltham, MA) is an *in vivo*, real time method to quantify protease enzyme activity, a marker of biocompatibility of implanted materials (34). Prosense is a cathepsin probe that becomes fluorescent after enzyme cleavage (35, 36). Neutrophils, along with monocytes and macrophages, release cathepsins during degranulation. Cathepsins are proteolytic enzymes that digest foreign material (37).

All animals were maintained on sterilized AIN-93G purified rodent diet (TD94045, Envigo, Indianapolis, Indiana) *ad libitum* to minimize the fluorescent background during quantification of inflammation using *in vivo* Imaging System (IVIS, Spectrum Instruments Imaging, Tuscon, Arizonia) imaging. The hindlimbs were shaved to minimize fluorescent background after injection. At postoperative days 6 and 13, 200 μL (8 nmol) of ProSense 750 FAST per rat was injected intravenously via the tail vein. At postoperative days 7 and 24 (24 h post-intravenous administration of the ProSense 750 FAST substrate, cleaved into a fluorescent tracking product by immune-secreted cathepsin enzymes), the rats were anesthetized using 3% isoflurane in oxygen and maintained at the same rate throughout the procedure. New grown hair was removed by Nair™ hair removal lotion (Church & Dwight Co., Inc., Ewing Township, New Jersey) and the rats were scanned by the IVIS (Xenogen, Caliper LifeScience, Waltham, MA) at the following settings: exposure = 7.50, binning = medium, FStop = 2, excitation = 605 ~ 640 nm and emission = 660 ~ 760 nm. Images were analyzed using Living Image Software (Perkin Elmer, Waltham, MA). The region of interest on the contralateral hindlimb was used for background subtraction during the signal quantification to remove fluorescent signal due to autofluorescence.

### Histological Processing for H&E and Masson Trichrome Staining

The gastrocnemius muscles, electrode and skin were excised and collected on postoperative day 14 and fixed overnight in 4% paraformaldehyde at 4°C. After fixation, the tissues were washed using 70% alcohol. The tissues were then paraffin embedded, sectioned and stained according to standard H&E and Masson trichrome histological methods at the Hope Babette Tang Histology Facility at the Koch Institute at the Massachusetts Institute of Technology.

### Immunofluorescence Staining and Microscopy

Following processing and embedding in paraffin blocks, sections (5–10 μm) were prepared by microtome (Leica Biosystems, Wetzlar, Germany). Prior to staining, samples were dewaxed and steamed for 45 min in antigen retrieval buffer (pH 6, Citra plus solution, Biogenex Laboratories, San Ramon, CA), and washed three times with 1X PBS prior to permeabilization for 5 min using 1% Triton X100. Samples were then blocked for 1 h using 1% bovine serum albumin (BSA) solution, and then incubated for 1 h with antibodies (1:200 dilution) for macrophage marker CD68-AF488 (Clone# Y1/82A, Cat# 333811, BioLegend, San Diego, CA) and Cy3-conjugated anti-mouse α-smooth muscle actin (fibrosis) (Sigma Aldrich, St. Louis, MO) in BSA. Samples were then rinsed and Prolong Antifade Gold mountant (Cat# P36930, ThermoFisher, Waltham, MA) was applied prior to cover slipping and sealing. Images were taken using an LSM 700-point scanning confocal microscope (Carl Zeiss Microscopy, Jena, Germany) equipped with 10X objectives.

### Quantitative Polymerase Chain Reaction (qPCR) Analysis

The following targets were chosen for gene expression to evaluate degree of inflammation or immune response against the electrode in the No Flap vs. Flap groups: CD68, α-SMactin and TNF-α. As previously described (21), RNA was isolated from samples of tissue including both the skin and underlying gastrocnemius muscle that were snap-frozen in liquid nitrogen following excision, using the TRIzol protocol (Invitrogen, Carlsbad, CA). All samples were normalized by loading 1 μg total RNA for reverse transcription using the High Capacity cDNA Reverse Transcription kit (Applied Biosystems, Foster City, CA). cDNA (1:20 dilution) was amplified by qPCR with the following primers: Rat CD68 (5′-GCCACAGTACAGTCTACCTTA-3′; reverse: 5′-AGAGATGAATTCTGCGCTGA-3′), Rat α-SMactin (5′-CGCTTCCGCTGCCCGGAGACC-3′; reverse: 5′-TATAGGTGGTTTCGTGGATGCCCGCC-3′), and Rat TNF-α (5′-CACGCTCTTCTGTCTACTGAACTTC-3′; reverse: 5′-GAGTGTGAGGGTCTGGGCCATG-3′). Signatures were normalized to rat (5′-ACCTTCTTGCAGCTCCTCCGTC-3′; reverse: 5′-CGGAGCCGTTGTCGACGACG-3′) β-actin. Samples were incubated for 10 min at 95°C followed by 40 cycles of 95°C for 15 sec and 60°C for 60 sec in a Roche 480 LightCycler. Results were analyzed by comparative C_T_ (ΔΔC_T_) method and are presented as RNA levels after normalization to β-actin.

### Statistical Analysis

Statistical comparisons were performed using GraphPad Prism 7.0 (Graph Pad software, Inc. La Jolla, California). Data are presented as mean+/–SEM. Values of *P* < 0.05 were considered statistically significant. For qPCR or IVIS, continuous data were analyzed for statistical significance by Mann–Whitney test.

## Results

### Postoperative Course and Gross Observation

**Figure 3:**
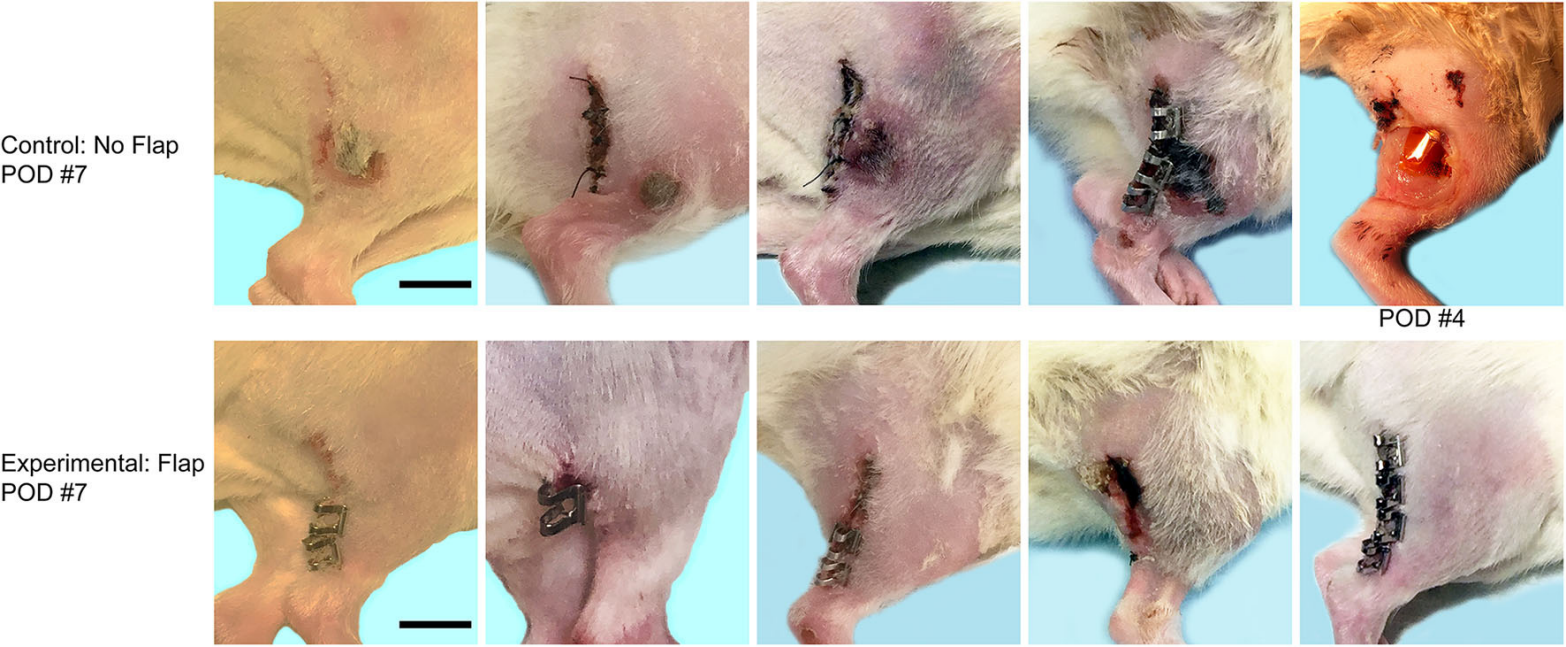
Top row shows wound after electrode implantation without flap on postoperative day (POD) 7. One animal which demonstrated complete wound dehiscence with extrusion of the electrode on POD 4, requiring immediate euthanasia. Bottom row shows incision after electrode implantation with flap on POD 7. Scale bar = 1 cm.

The mean time for surgical procedures was 60 min. After surgery, all 10 animals recovered without incident and were eating and drinking by postoperative day 1. On postoperative day 4, one animal in the No Flap group demonstrated rejection of the epimysial implant and complete wound dehiscence with complete erosion of the overlying skin, requiring euthanasia of the animal (Figure 3). On postoperative day 7, ulcerative lesions were observed at the implant site among the remaining 4 animals in the No Flap group whereas there were no ulcerative lesions present among the 5 animals in the Flap group.

### Evaluation of Histology and Immunofluorescence

**Figure 4:**
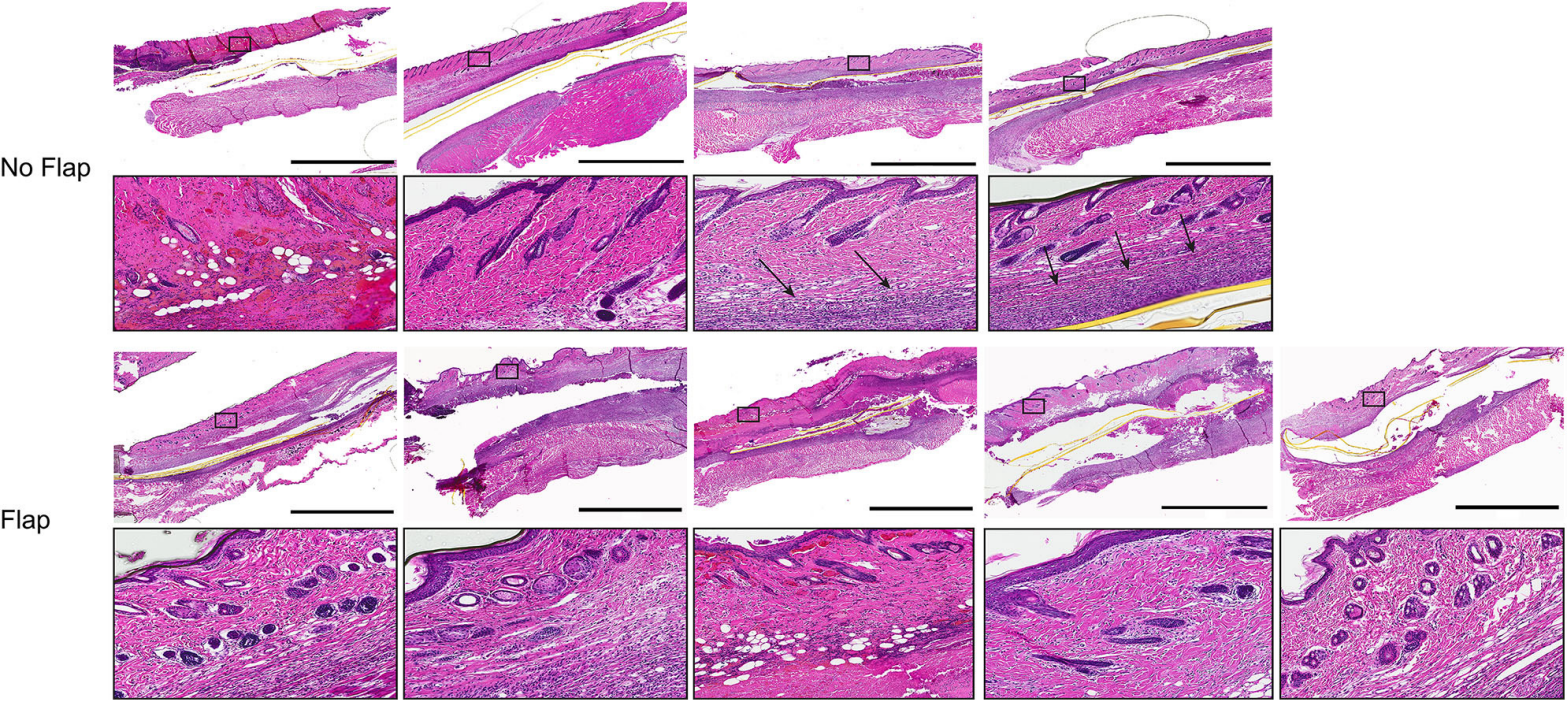
H&E stained histological sections of excised gastrocnemius muscles and overlying skin at postoperative day 14 after 10 days of therapeutic functional electrical stimulation after peripheral nerve injury. Higher magnification regions of interest allow for observation of inflammatory cell infiltration within the dermal layer overlying the electrodes (black arrows). Scale bars = 3 mm.

**Figure 5:**
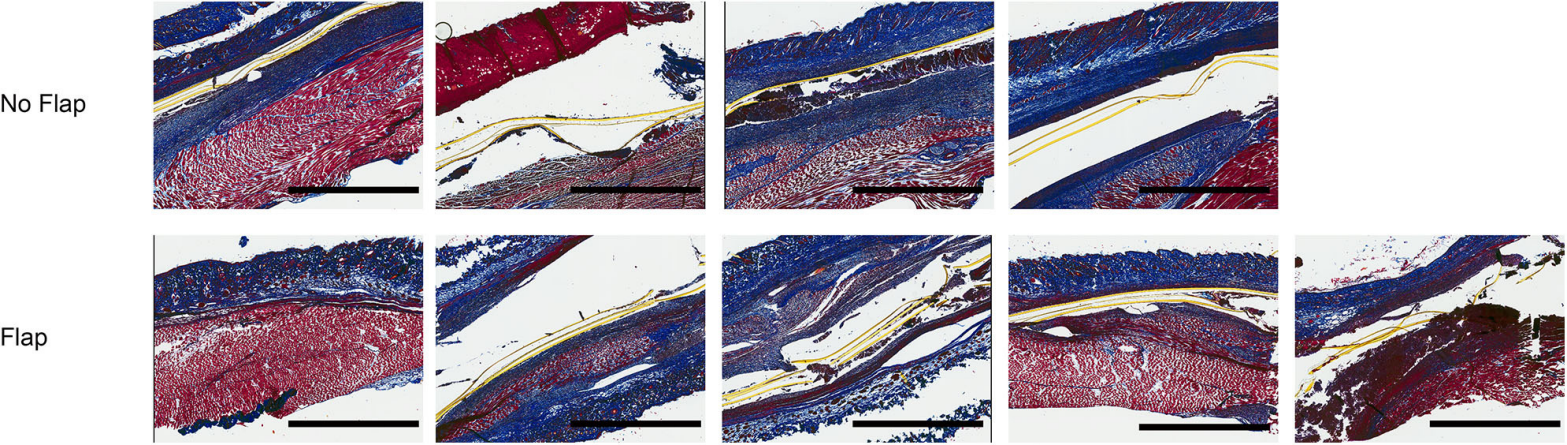
Masson trichrome stained sections of excised gastrocnemius muscles and overlying skin at postoperative day 14 after 10 days of therapeutic functional electrical stimulation after critical peripheral nerve injury. Staining highlights thick fibrotic capsule formation surrounding electrode, particularly in the “No Flap” group. Scale bars = 2 mm.

**Figure 6:**
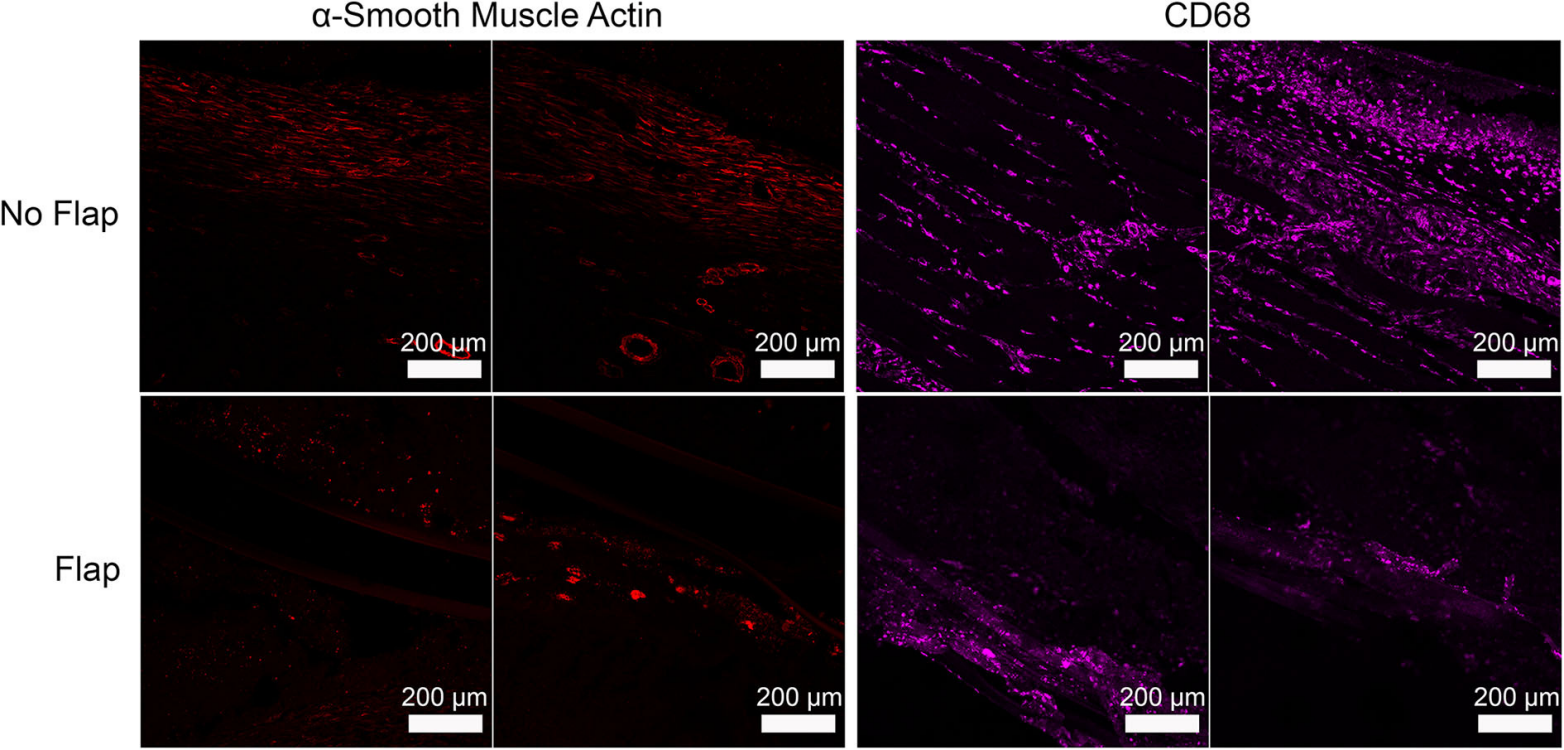
Representative immunofluorescence staining of α-smooth muscle actin (shown as red) and CD68 (shown as green) illustrating foreign body response to implanted electrode that is more concentrated at the site of the electrode in the “Flap” group whereas in the “No Flap” group, the foreign body response infiltrates throughout the muscle fibers.

Histological sections of excised gastrocnemius muscle, biceps femoris muscle flap (if present) and overlying skin were stained with hematoxylin and eosin (H&E) to assess electrode biocompatibility and immune cell infiltration (Figure 4). Magnified sections of the dermis overlying the electrode are shown. In the “No Flap” group, the architecture of the dermal and epidermal layers is largely replaced by scar relative to the “Flap” group. Masson trichrome staining (MTS) was also performed to evaluate collagen organization within the granulation tissue and scar surrounding the electrode. MTS shows thick fibrotic capsule formation surrounding the electrode, particularly in the “No Flap” group (Figure 5). Figure 6 shows representative confocal immunofluorescence for markers of foreign body response including CD68 and α-smooth muscle actin. In the “Flap” group, foreign body response was primarily concentrated at the site of the electrode whereas in the “No Flap” group, there was infiltration throughout the muscle fibers which may contribute to implant failure in the “No Flap” group.

### Quantification of Immune Response to Implanted Electrodes

**Figure 7:**
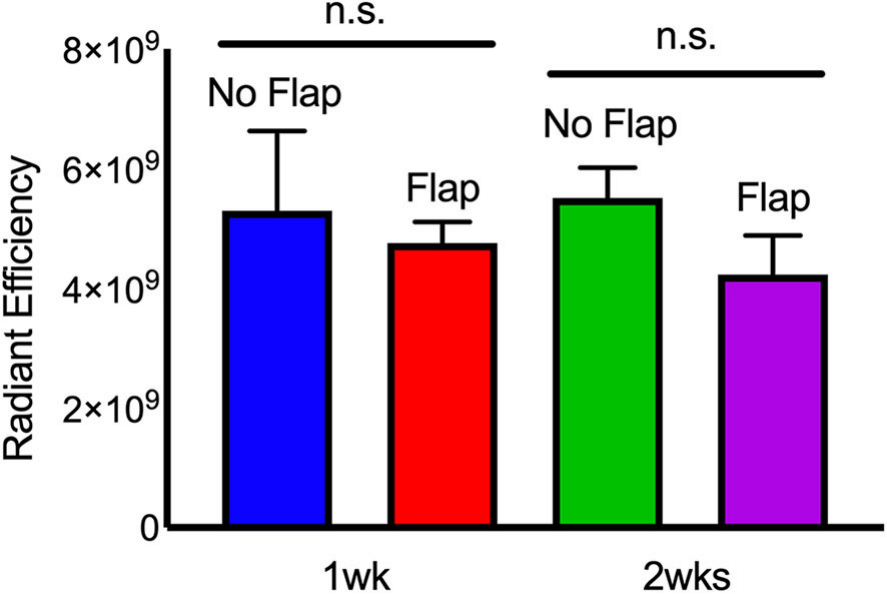
IVIS of “Flap” vs. “No Flap” after postoperative day 7 and 14. Quantification of IVIS signals and statistical analysis showing significantly reduced inflammation at 1 week. n.s. indicates not statistically significant compared to the “No Flap” group at the level of *p* < 0.05 using Mann–Whitney test. *N* = 5 rats/group.

**Figure 8:**
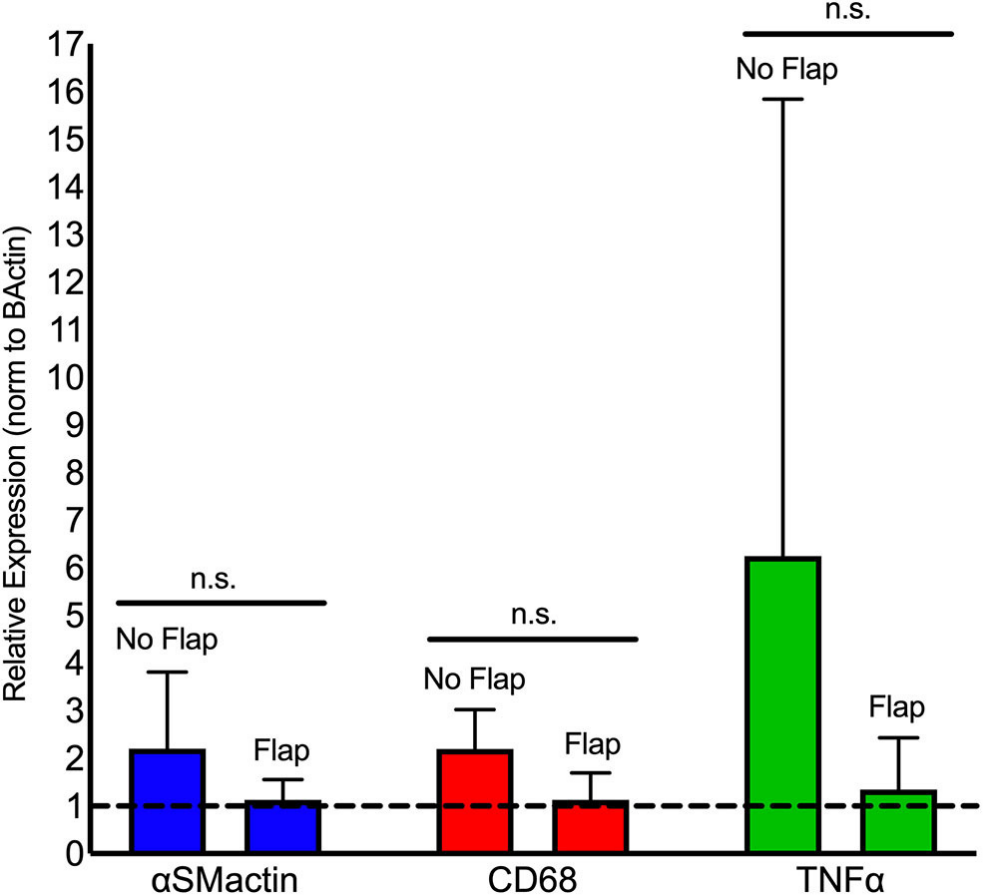
qPCR analysis of fibrotic (αSMactin), macrophage (CD68), and acute phase reactant (TNFα) markers present within gastrocnemius muscle directly adjacent to the electrode relative to rat beta-actin levels (dashed horizontal line). Statistical analysis shows a significant reduction in mean macrophage populations in the Flap group relative to the No Flap group. n.s. indicates not statistically significant compared to the No Flap group at the level of *p* < 0.05 using Mann–Whitney test. *N* = 5 rats/group.

Immune cell activity at the site of electrode implantation in both the Flap and No Flap groups was quantified using ProSense, a fluorescent indicator of immune-associated inflammation (34). IVIS at 7- and 14-days post-implantation showed decreases in inflammation in the Flap group although there were no statistically significant differences (Figure 7). The animals were euthanized on postoperative day 14 (*n* = 5 with Flap and *n* = 4 No Flap) and the gastrocnemius muscle, overlying skin and/or biceps femoris flap were excised for qPCR. An increased recruitment of αSM-actin^+^ fibroblasts, CD68^+^ macrophages and TNF-α acute phase reactants were evidenced in the No Flap group; however, no statistically significant difference was evident (Figure 8). Increased expression of these markers of inflammation correlate with increased scarring around the electrode and breakdown of the skin overlying the electrode as evident on histology and gross examination.

## Discussion

Despite being comprised of biocompatible materials, the implanted electrode for delivery of FES to treat muscle atrophy causes skin ulceration and dehiscence. Skin breakdown is a common complication of electrode and device implantation, and FES via implanted electrodes may enhance these deleterious effects. Primary predisposing factors for implant exposure include inadequate soft tissue coverage, soft tissue thinning over time and scar dehiscence over edges of the implant. When skin breakdown becomes so severe that the implant is exposed, the implant must often be removed entirely. Skin ulceration and subsequent exposure of implantable electrical devices results in bacterial contamination and subsequent infection, which may increase mortality significantly (38). Often, the tissue around the exposed implant and the implant itself must be removed entirely to control infections, followed by targeted antibiotic treatment for a minimum of 6 weeks. Secondary operations may be required for delayed repair, requiring general anesthesia and increasing the risk of patient morbidity. Management procedures for implant exposure without implant removal include antibiotics, irrigation with n-acetylcysteine solution (39), capsulectomy, dermal grafts (40) and muscle flap coverage (41) have been largely unsuccessful in preventing infection relapse.

Given the suboptimal treatments available to manage wound dehiscence and subsequent implant exposure, several techniques have been developed for prevention of this complication. Special attention has been given to improving closure technique since achieving appropriate skin approximation and minimal wound tension is key for healing and preventing infections (42, 43). New adhesive strips are being investigated as an alternate to sutures for closure of surgical incisions overlying implanted materials to prevent dehiscence (44). The Zip® Surgical Skin Closure system (Zipline Medical Inc., Campell, CA) is a hydrocolloid adhesive tape that is applied on each side of the incision and adjustable plastic ratcheting pieces are tightened to close the superficial skin. This device has been described for incisions of cardiac implantable electronic devices (45, 46). However, the efficacy of adhesive strips in preventing wound dehiscence and infections has not been well-established.

Negative pressure wound therapy (NPWT) has been described as a tool to treat dehiscence and surgical site infections (SSIs). Recently, NPWT has been described for its role in preventing dehiscence and SSIs of closed incisions following instrumented spinal fusion surgery (47). During incisional NPWT, the wound is covered with a moist sterile gauze, sealed with an adhesive tape and connected to a vacuum pump using a drainage hose. The mechanism by which incisional NPWT facilitates wound healing is through maintenance of a sterile field, decreased tension across the incision, removal of fluid from the wound edges, reduced pathogen burden and promotion of granulation tissue via angiogenesis (48–50). Naylor et al. (47) showed prevention of increased rates of dehiscence and SSI among patients undergoing lumbar fusion through an anterior approach and there are currently two ongoing clinical trials to further investigate the efficacy of NPWT in spinal fusion surgery (NCT03820219 and NCT03632005).

Another technique for prevention of wound dehiscence and implant exposure is the use of prophylactic muscle flaps. Vascularized muscle and skin flaps have been widely used to treat exposed implants during revision surgeries. For instance, exposed pacemakers have been treated by utilizing a modified rotation skin flap over the area of scar dehiscence (51). This technique successfully prevents pacemaker infection and prevents replacement of the device completely. Muscle flaps have become important for reconstruction of soft tissue defects because they bring a rich vascular supply to the wound and demonstrate superior resistance to infection (52). As an example, pedicled sub-mammary intercostal perforator flaps have been successfully used to treat exposure of titanium-coated polypropylene mesh used in breast reconstruction (53). Using vascularized muscle flaps as a prophylactic measure before exposure of an implant is a relatively new technique. The vascular supply created by a local flap may add robustness to muscle treated by epimysial FES through the enhanced mass transport of nutrients, waste and breakdown products. Subpectoral placement of a pacemaker is one example of a muscle flap used as a prophylactic measure to prevent wound breakdown as a primary procedure as opposed to a reconstruction during a second intervention after breakdown or infection has already occurred. Prophylactic soft tissue augmentation with a perforator flap has been described during total knee arthroplasties for prevention of excessive scarring as well as prevention of wound breakdown and periprosthetic infection (54). Furthermore, the presence of a vascularized muscle flap may provide a platform for angiogenesis accelerating nerve regeneration (55, 56).

Some disadvantages of using vascularized muscle flaps include donor deficit, longer learning curve for surgeons as well as discomfort and spasm in some cases (57, 58). Donor site morbidity is a significant limitation of muscle flaps, leading to functional deficit in some cases. Appropriate compensation for any functional deficit must be demonstrated when choosing a muscle to use as a flap (59–61).

The rat biceps femoris (BF) muscle flap was first described by Akyurek et al. (29). This anatomic study revealed that the main vascular supply to the BF muscle was the caudal femoral branch of the popliteal vessels. A subsequent study (62) showed four vessels supplying the BF muscle flap: the most superior vessel arising from the lateral superior genucular artery, which is the first branch of the caudal femoral artery, and the other three originate from the trunk of the caudal femoral artery. All of these vessels are small and located within the popliteal fossa fat pad. In order for a muscle to be a good candidate for a flap model, the muscle should have a readily identifiable vascular pedicle and should be easily harvested. The location of the BF muscle vascular pedicle is consistent and there is no risk of autocanalization of the flap since it is located dorsally. Other established muscular flaps have included the gastrocnemius, latissimus, serratus anterior, gracilis, thigh adductor, abdominal wall, rectus abdominis, abdominal wall, pectoralis major, and quadriceps flaps (63).

Here, we described a novel use of this vascularized muscle flap and with it the blood supply to secrete growth factors that accelerate wound healing to prevent skin breakdown during FES of the gastrocnemius muscle to treat muscle atrophy during peripheral nerve injury. The strength of this study is that we demonstrated this procedure avoids the need for secondary intervention, preventing delays in treatment and unnecessary morbidity. “Regarding the potential clinical translatability of these findings, there are several critical considerations. First, the present results cannot be directly extrapolated to humans. The procedure described here utilizing the BF muscle as a vascularized flap is unique to the anatomy of rats and currently there is no clear correlate in the anatomy of humans although the BF muscle turnover flap has been described for reconstruction of ischial pressure ulcers and abscesses (64, 65). Second, the number of animals in this pilot study was small (*n* = 10) and, given the feasibility of this procedure demonstrated in this pilot study, a larger and longer-term follow-up study is necessary to validate the results described here. Furthermore, this surgery also increases operative time due to the prolonged dissection and handling required to transpose the BF muscle. Complications that may occur at an increased rate with this procedure include hematoma or seroma formation. While these complications were not observed in this study, such complications may be prevented by careful hemostasis of the muscle flap during the procedure and treatment of these complications includes drainage, lavage and re-suturing. Compression over the dressing may also prevent these complications in the postoperative setting. This technique may be considered in future studies of implants in the rat hindlimb.

## Data Availability Statement

The raw data supporting the conclusions of this article will be made available by the authors, without undue reservation.

## Ethics Statement

The animal studies were reviewed and approved by the Massachusetts Institute of Technology's Institutional Animal Care and Use Committee (IACUC).

## Author Contributions

MM and JD performed all experiments, data interpretation, and manuscript writing. All authors contributed to manuscript revision, read, and approved the submitted version.

## Conflict of Interest

For a list of entities with which RL is involved, compensated or uncompensated, see www.dropbox.com/s/yc3xqb5s8s94v7x/Rev%20Langer%20COI.pdf?dl=0. The remaining authors declare that the research was conducted in the absence of any commercial or financial relationships that could be construed as a potential conflict of interest.

## References

[1] Bentolila V Nizard R Bizot P Sedel L. Complete traumatic brachial plexus palsy. Treatment and outcome after repair. J Bone Joint Surg Am. (1999) 81:20–8. 10.2106/00004623-199901000-00004 9973050

[2] Gordon T Tyreman N Raji MA. The basis for diminished functional recovery after delayed peripheral nerve repair. J Neurosci. (2011) 31:5325–34. 10.1523/JNEUROSCI.6156-10.2011 21471367 PMC6622714

[3] Stoll G Muller HW. Nerve injury, axonal degeneration and neural regeneration: basic insights. Brain Pathol. (1999) 9:313–25. 10.1111/j.1750-3639.1999.tb00229.x 10219748 PMC8098499

[4] Willand MP Chiang CD Zhang JJ Kemp SW Borschel GH Gordon T. Daily electrical muscle stimulation enhances functional recovery following nerve transection and repair in rats. Neurorehabil Neural Repair. (2015) 29:690–700. 10.1177/1545968314562117 25505222

[5] Mcavoy M Tsosie JK Vyas KN Khan OF Sadtler K Langer R. Flexible multielectrode array for skeletal muscle conditioning, acetylcholine receptor stabilization and epimysial recording after critical peripheral nerve injury. Theranostics. (2019) 9:7099–107. 10.7150/thno.35436 31660089 PMC6815960

[6] Al-Majed AA Brushart TM Gordon T. Electrical stimulation accelerates and increases expression of BDNF and trkB mRNA in regenerating rat femoral motoneurons. Eur J Neurosci. (2000) 12:4381–90. 10.1111/j.1460-9568.2000.01341.x 11122348

[7] Al-Majed AA Tam SL Gordon T. Electrical stimulation accelerates and enhances expression of regeneration-associated genes in regenerating rat femoral motoneurons. Cell Mol Neurobiol. (2004) 24:379–402. 10.1023/B:CEMN.0000022770.66463.f7 15206821 PMC11529956

[8] Hultman CS Jones GE Losken A Seify H Schaefer TG Zapiach LA. Salvage of infected spinal hardware with paraspinous muscle flaps: anatomic considerations with clinical correlation. Ann Plast Surg. (2006) 57:521–8. 10.1097/01.sap.0000226931.23076.a7 17060733

[9] English AW Schwartz G Meador W Sabatier MJ Mulligan A. Electrical stimulation promotes peripheral axon regeneration by enhanced neuronal neurotrophin signaling. Dev Neurobiol. (2007) 67:158–72. 10.1002/dneu.20339 17443780 PMC4730384

[10] Zhang X Xin N Tong L Tong XJ. Electrical stimulation enhances peripheral nerve regeneration after crush injury in rats. Mol Med Rep. (2013) 7:1523–7. 10.3892/mmr.2013.1395 23545781

[11] Nix WA. The effect of low-frequency electrical stimulation on the denervated extensor digitorum longus muscle of the rabbit. Acta Neurol Scand. (1982) 66:521–8. 10.1111/j.1600-0404.1982.tb03138.x 7148394

[12] Dow DE Cederna PS Hassett CA Kostrominova TY Faulkner JA Dennis RG. Number of contractions to maintain mass and force of a denervated rat muscle. Muscle Nerve. (2004) 30:77–86. 10.1002/mus.20054 15221882

[13] Kern H Boncompagni S Rossini K Mayr W Fano G Zanin ME. Long-term denervation in humans causes degeneration of both contractile and excitation-contraction coupling apparatus, which is reversible by functional electrical stimulation (FES): a role for myofiber regeneration? J Neuropathol Exp Neurol. (2004) 63:919–31. 10.1093/jnen/63.9.919 15453091

[14] Ashley Z Salmons S Boncompagni S Protasi F Russold M Lanmuller H. Effects of chronic electrical stimulation on long-term denervated muscles of the rabbit hind limb. J Muscle Res Cell Motil. (2007) 28:203–17. 10.1007/s10974-007-9119-4 17906933

[15] Ashley Z Sutherland H Russold MF Lanmuller H Mayr W Jarvis JC. Therapeutic stimulation of denervated muscles: the influence of pattern. Muscle Nerve. (2008) 38:875–86. 10.1002/mus.21020 18563723

[16] Park SJ Park JS Yang HN Yi SW Kim CH Park KH. Neurogenesis is induced by electrical stimulation of human mesenchymal stem cells co-cultured with mature neuronal cells. Macromol Biosci. (2015) 15:1586–94. 10.1002/mabi.201500115 26183918

[17] Von Roth P Winkler T Rechenbach K Radojewski P Perka C Duda GN. Improvement of contraction force in injured skeletal muscle after autologous mesenchymal stroma cell transplantation is accompanied by slow to fast fiber type shift. Transfus Med Hemother. (2013) 40:425–30. 10.1159/000354127 24474893 PMC3901591

[18] Di Filippo ES Mancinelli R Marrone M Doria C Verratti V Toniolo L. Neuromuscular electrical stimulation improves skeletal muscle regeneration through satellite cell fusion with myofibers in healthy elderly subjects. J Appl Physiol. (2017) 123:501–12. 10.1152/japplphysiol.00855.2016 28572500

[19] Khodabukus A Prabhu N Wang J Bursac N. *In vitro* tissue-engineered skeletal muscle models for studying muscle physiology and disease. Adv Healthc Mater. (2018) 7:e1701498. 10.1002/adhm.201701498 29696831 PMC6105407

[20] Guo L Ma M Zhang N Langer R Anderson DG. Stretchable polymeric multielectrode array for conformal neural interfacing. Adv Mater. (2014) 26:1427–33. 10.1002/adma.201304140 24150828 PMC4047984

[21] Farah S Doloff JC Muller P Sadraei A Han HJ Olafson K. Long-term implant fibrosis prevention in rodents and non-human primates using crystallized drug formulations. Nat Mater. (2019) 18:892–904. 10.1038/s41563-019-0377-5 31235902 PMC7184801

[22] Srinivasan S Vyas K Mcavoy M Calvaresi P Khan OF Langer R. Polyimide electrode-based electrical stimulation impedes early stage muscle graft regeneration. Front Neurol. (2019) 10:252. 10.3389/fneur.2019.00252 30967830 PMC6438882

[23] Subbaroyan J Martin DC Kipke DR. A finite-element model of the mechanical effects of implantable microelectrodes in the cerebral cortex. J Neural Eng. (2005) 2:103–13. 10.1088/1741-2560/2/4/006 16317234

[24] Woods WA Jr Fusillo SJ Trimmer BA. Dynamic properties of a locomotory muscle of the tobacco hornworm Manduca sexta during strain cycling and simulated natural crawling. J Exp Biol. (2008) 211:873–82. 10.1242/jeb.006031 18310113

[25] Richardson RR Jr Miller JA Reichert WM. Polyimides as biomaterials: preliminary biocompatibility testing. Biomaterials. (1993) 14:627–35. 10.1016/0142-9612(93)90183-3 8399958

[26] Lesavoy MA Dubrow TJ Wackym PA Eckardt JJ. Muscle-flap coverage of exposed endoprostheses. Plast Reconstr Surg. (1989) 83:90–9. 10.1097/00006534-198901000-00017 2909082

[27] Tan KJ Lim CT Lim AYT. The use of muscle flaps in the salvage of infected exposed implants for internal fixation. J Bone Joint Surg Br. (2010) 92, 401–5. 10.1302/0301-620X.92B3.22115 20190312

[28] Fischer JP Nelson JA Mirzabeigi MN Wang GJ Foley PJ III Wu LC Woo EY. Prophylactic muscle flaps in vascular surgery. J Vasc Surg. (2012) 55:1081–6. 10.1016/j.jvs.2011.10.110 22209610

[29] Akyurek M Safak T Manavbasi I Kecik A. A rat musculocutaneous flap model: the biceps femoris musculocutaneous flap. Ann Plast Surg. (2000) 45:305–12. 10.1097/00000637-200045030-00014 10987534

[30] Ozkan O Coskunfirat OK Ozgentas HE. A new experimental flap model: free muscle perforator flap. Ann Plast Surg. (2003) 51:603–6. 10.1097/01.sap.0000095655.59282.23 14646658

[31] Ozkan O Coskunfirat OK Ozgentas HE Dikici MB. New experimental flap model in the rat: free flow-through epigastric flap. Microsurgery. (2004) 24:454–8. 10.1002/micr.20063 15499551

[32] Ozkan O Coskunfirat OK Dogan O Ozgentas HE. A reverse-flow composite flap in the rat. J Plast Reconstr Aesthet Surg. (2007) 60:556–62. 10.1016/j.bjps.2006.01.034 17399666

[33] Willand MP Holmes M Bain JR Fahnestock M De Bruin H. Determining the effects of electrical stimulation on functional recovery of denervated rat gastrocnemius muscle using motor unit number estimation. Conf Proc IEEE Eng Med Biol Soc. (2011) 2011:1977–80. 10.1109/IEMBS.2011.6090557 22254721 PMC3413723

[34] Bratlie KM Dang TT Lyle S Nahrendorf M Weissleder R Langer R. Rapid biocompatibility analysis of materials via *in vivo* fluorescence imaging of mouse models. PLoS ONE. (2010) 5:e10032. 10.1371/journal.pone.0010032 20386609 PMC2850367

[35] Faurschou M Borregaard N. Neutrophil granules and secretory vesicles in inflammation. Microbes Infect. (2003) 5:1317–27. 10.1016/j.micinf.2003.09.008 14613775

[36] Lominadze G Powell DW Luerman GC Link AJ Ward RA Mcleish KR. Proteomic analysis of human neutrophil granules. Mol Cell Proteomics. (2005) 4:1503–21. 10.1074/mcp.M500143-MCP200 15985654

[37] Vinay K Abbas AK Fauston N Aster JC Robbins and Cotran Pathologic Basis of Disease Saunders. Philadelphia, PA: Saunders (2009).

[38] Le KY Sohail MR Friedman PA Uslan DZ Cha SS Hayes DL. Impact of timing of device removal on mortality in patients with cardiovascular implantable electronic device infections. Heart Rhythm. (2011) 8:1678–85. 10.1016/j.hrthm.2011.05.015 21699855

[39] Zhao T Liu Y. N-acetylcysteine inhibit biofilms produced by *Pseudomonas aeruginosa*. BMC Microbiol. (2010) 10:140. 10.1186/1471-2180-10-140 20462423 PMC2882372

[40] Rudolph R Smith MR Curtis GP. Salvage of pacemakers and automatic implantable cardioverter-defibrillators using dermis grafts. Ann Thorac Surg. (2011) 91:452–6. 10.1016/j.athoracsur.2010.10.004 21256289

[41] Toia F D'arpa S Cordova A Moschella F. Exposed subcutaneous implantable devices: an operative protocol for management and salvage. Plast Reconstr Surg Glob Open. (2015) 3:e343. 10.1097/01.GOX.0000464837.59870.6f 26034650 PMC4448718

[42] Wa A Manual on Control of Infection in Surgical Patients. Philadelphia, PA: Lippincott (1984).

[43] Imai K. Perioperative management for the prevention of bacterial infection in cardiac implantable electronic device placement. J Arrhythm. (2016) 32:283–6. 10.1016/j.joa.2015.06.007 27588150 PMC4996848

[44] Gkegkes ID Mavros MN Alexiou VG Peppas G Athanasiou S Falagas ME. Adhesive strips for the closure of surgical incisional sites: a systematic review and meta-analysis. Surg Innov. (2012) 19:145–55. 10.1177/1553350611418989 21926099

[45] Lalani GG Schricker AA Salcedo J Hebsur S Hsu J Feld G. Cardiac device implant skin closure with a novel adjustable, coaptive tape-based device. Pacing Clin Electrophysiol. (2016) 39:1077–82. 10.1111/pace.12926 27470060

[46] Koerber SM Loethen T Turagam M Payne J Weachter R Flaker G. Noninvasive tissue adhesive for cardiac implantable electronic device pocket closure: the TAPE pilot study. J Interv Card Electrophysiol. (2019) 54:171–6. 10.1007/s10840-018-0457-5 30324225

[47] Naylor RM Gilder HE Gupta N Hydrick TC Labott JR Mauler DJ. Effects of negative pressure wound therapy on wound dehiscence and surgical site infection following instrumented spinal fusion surgery-A single surgeon's experience. World Neurosurg. (2020) 137:e257–62. 10.1016/j.wneu.2020.01.152 32004742 PMC8063507

[48] Orgill DP Manders EK Sumpio BE Lee RC Attinger CE Gurtner GC. The mechanisms of action of vacuum assisted closure: more to learn. Surgery. (2009) 146:40–51. 10.1016/j.surg.2009.02.002 19541009

[49] Karlakki S Brem M Giannini S Khanduja V Stannard J Martin R. Negative pressure wound therapy for managementof the surgical incision in orthopaedic surgery: a review of evidence and mechanisms for an emerging indication. Bone Joint Res. (2013) 2:276–84. 10.1302/2046-3758.212.2000190 24352756 PMC3884878

[50] Ousey KJ Atkinson RA Williamson JB Lui S. Negative pressure wound therapy (NPWT) for spinal wounds: a systematic review. Spine J. (2013) 13:1393–405. 10.1016/j.spinee.2013.06.040 23981819

[51] Tiwari R Marwah S Roy A Singhal M. Novel technique to manage pacemaker exposure with buried flap reconstruction: case series. Heart Asia. (2019) 11:e011086. 10.1136/heartasia-2018-011086 30728862 PMC6340535

[52] Mathes SJ Alpert BS Chang N. Use of the muscle flap in chronic osteomyelitis: experimental and clinical correlation. Plast Reconstr Surg. (1982) 69:815–29. 10.1097/00006534-198205000-00018 7071227

[53] De Riggi MA Rocco N Gherardini G Esposito E D'aiuto M. Management of implant exposure in one-stage breast reconstruction using titanium-coated polypropylene mesh: sub-mammary intercostal perforator flap. Aesthetic Plast Surg. (2016) 40:896–900. 10.1007/s00266-016-0720-z 27766402

[54] Azoury SC Stranix JT Piper M Kovach SJ Hallock GG. Attributes of perforator flaps for prophylatic soft tissue augmentation prior to definitive total knee arthroplasty. J Reconstr Microsurg. (2019). 10.1055/s-0039-3401847. [Epub ahead of print]. 31877565

[55] Pan HC Chang MH Sheu ML Chen CJ Sheehan J. Increased angiogenesis by the rotational muscle flap is crucial for nerve regeneration. PLoS ONE. (2019) 14:e0217402. 10.1371/journal.pone.0217402 31181105 PMC6557495

[56] Choi J Kim DS Kim J Jeong W Lee HW Park SW. Better nerve regeneration with distally based fascicular turnover flap than with conventional autologous nerve graft in a rat sciatic nerve defect model. J Plast Reconstr Aesthet Surg. (2020) 73:214–21. 10.1016/j.bjps.2019.09.031 31690543

[57] Fayman MS Chait LA Orak F. A subpectoral pocket in the management of a patient with impending extrusion of a pulse generator. Plast Reconstr Surg. (1986) 78:182–5. 10.1097/00006534-198608000-00006 3725964

[58] Bonawitz SC. Management of exposure of cardiac pacemaker systems. Ann Plast Surg. (2012) 69:292–5. 10.1097/SAP.0b013e31822350cc 21734547

[59] Harii K Ohmori K Torii S. Free gracilis muscle transplantation, with microneurovascular anastomoses for the treatment of facial paralysis. A preliminary report. Plast Reconstr Surg. (1976) 57:133–43. 10.1097/00006534-197602000-00001 1250883

[60] Forrest C Boyd B Manktelow R Zuker R Bowen V. The free vascularised iliac crest tissue transfer: donor site complications associated with eighty-two cases. Br J Plast Surg. (1992) 45:89–93. 10.1016/0007-1226(92)90163-R 1562853

[61] Gardetto A Raschner C Schoeller T Pavelka ML Wechselberger G. Rectus femoris muscle flap donor-site morbidity. Br J Plast Surg. (2005) 58:175–82. 10.1016/j.bjps.2004.08.001 15710112

[62] Zhu H Xie F Shen L Li Q. Blood supply of the rat biceps femoris musculocutaneous flap. J Reconstr Microsurg. (2015) 31:160–2. 10.1055/s-0034-1381956 25226083

[63] Zhang F Sones WD Lineaweaver WC. Microsurgical flap models in the rat. J Reconstr Microsurg. (2001) 17:211–21. 10.1055/s-2001-14353 11336153

[64] Demirseren ME Ceran C Aksam B Demiralp CO. Clinical experience with the combination of a biceps femoris muscle turnover flap and a posterior thigh fasciocutaneous hatchet flap for the reconstruction of ischial pressure ulcers. Ann Plast Surg. (2016) 77:93–6. 10.1097/SAP.0000000000000290 25057917

[65] Schmidt I A devasting course of an iliopsoas muscle abscess subsequently leading to septic shock, septic hip arthritis, and extended gluteal soft tissue necroses in an elderly immunocompromised patient with multiple carcinomas: a case report and brief review of literature. Open Orthop J. (2018) 12:180–9. 10.2174/1874325001812010180 29997705 PMC5997861

